# Supplementary material for: Nuclear genome of Bulinus truncatus, an intermediate host of the carcinogenic human blood fluke Schistosoma haematobium
Source: Nat Commun. 2022 Feb 21;13:977. doi: 10.1038/s41467-022-28634-9 (PMC8861042; doi:10.1038/s41467-022-28634-9)
Supplement: Supplementary file 1 — Supplementary Information [file 41467_2022_28634_MOESM1_ESM.pdf]

Supplementary Table 1 | Genomic DNA libraries constructed for the sequencing of the nuclear genome of *Bulinus truncatus*.

	Number of sequences	Number of bases	N50	Read length	Genome coverage	Publication
DNA libraries						
Short read sequencing						
DNA - 500 base R1	437,728,763	64,274,150,630	na	150	52.61	This study
DNA - 500 base R2	437,728,763	64,274,150,630	na	150	52.61	This study
Long read sequencing						
Oxford Nanopore	2,860,324	11,440,314,362	12,657	na	9.36	This study
Long range sequencing						
Hi-C DNA library R1	238,668,351	23,866,835,100	na	100	19.53	This study
Hi-C DNA library R2	238,668,351	23,866,835,100	na	100	19.53	This study
RNAseq libraries						
Short read sequencing						
RNA ~300 base R1	38,186,011	5,727,901,650	na	150		Reference 23
RNA ~300 base R2	38,186,011	5,727,901,650	na	150		Reference 23
Long read sequencing						
Long RNAseq (direct RNA)	2,109,546	1,731,420,341	983	na		Reference 23
Long RNAseq (cDNA)	16,236,365	9,486,921,711	783	na		This study

Supplementary Table 2 | Summary of Hi-C reads that mapped to the draft genome (Btru.v1) of *Bulinus truncatus* and inferred Hi-C contacts.

Feature	Total number of reads (% of total)
Sequenced Read Pairs	216,235,256
Normal Paired	170,662,488 (78.92%)
Chimeric Paired	3,084,396 (1.43%)
Chimeric Ambiguous	1,239,409 (0.57%)
Unmapped	41,248,963 (19.08%)
Ligation Motif Present	5,341,194 (2.47%)
Alignable (Normal+Chimeric Paired)	173,746,884 (80.35%)
Unique Reads	82,804,233 (38.29%)
PCR Duplicates	90,795,714 (41.99%)
Optical Duplicates	146,937 (0.07%)
Library Complexity Estimate	100,827,346
Intra-fragment Reads	50,861,760 (23.52% / 61.42%)
Below MAPQ Threshold	23,643,688 (10.93% / 28.55%)
Hi-C Contacts	8,298,785 (3.84% / 10.02%)
Ligation Motif Present	814,837 (0.38% / 0.98%)
3' Bias (Long Range)	61% - 39%
Pair Type %(L-I-O-R)	25% - 26% - 24% - 25%
Inter-chromosomal	908,642 (0.42% / 1.10%)
Intra-chromosomal	7,390,143 (3.42% / 8.92%)
Short Range (<20Kb)	7,060,604 (3.27% / 8.53%)
Long Range (>20Kb)	329,515 (0.15% / 0.40%)

Supplementary Table 3 | Repeat elements in the draft genome (Btru.v1) of *Bulinus truncatus*.

Feature	Number of elements	Total bases	Percentage of genome	Elements per 500 kb: Mean	Median	Standard deviation	Genomic regions with highest number of repeat elements
Retrotransposons	173413	74927629	6.13%				
LINEs:	44451	16508637	1.35%	16.5	16	9.4	HiC_scaffold_188_500000-1000000 (157); HiC_scaffold_52_2500000-3000000 (140); HiC_scaffold_188_1000000-1500000 (113); HiC_scaffold_25_6000000-6500000 (100); HiC_scaffold_191_500000-1000000 (82)
RTE/Bov-B	24602	9867938	0.81%				
R1/Jockey	5871	2549585	0.21%				
R1 Unclassified	1010	1445885	0.12%				
R1/LOA	7951	1140426	0.09%				
R4/Dong	2094	470447	0.04%				
L1-Tx1	368	273087	0.02%				
LINE Unclassified	2555	761269	0.06%				
LTR elements:	128962	58418992	4.78%	47.8	46	21.6	HiC_scaffold_34_5000000-5500000 (174); HiC_scaffold_309_0-500000 (163); HiC_scaffold_10_4000000-4500000 (153); HiC_scaffold_591_0-500000 (152); HiC_scaffold_31_6000000-6500000 (151)
Ty1/Copia	728	501495	0.04%				
Gypsy/DIRS1	24758	14847697	1.22%				
BEL/Pao	458	353757	0.03%				
LTR Unclassfied	103018	42716043	3.50%				
DNA transposons	1017352	289274154	23.68%	375.8	397	115.4	HiC_scaffold_252_500000-955227 (1062); HiC_scaffold_26_0-500000 (897); HiC_scaffold_58_1000000-1500000 (773); HiC_scaffold_620_0-486623 (695); HiC_scaffold_8_7500000-8000000 (682)
TIR/hAT hobo-Activator (DTA)	424483	139583103	11.42%				
hAT-hATm	18688	16172625	1.32%				
hAT-Tip100	19235	9780900	0.80%				
hAT-Charlie	11989	3999790	0.33%				

hAT-Ac	6733	3504018	0.29%
hAT-hobo	1417	538855	0.04%
/hAT-Blackjack	73	14359	0.00%
hAT Unclassified	366348	105572556	8.64%
TIR/Mutator (DTM)	192719	47519892	3.89%
MuDR_Mutator	250	157677	0.01%
Mutator unclassified	192469	47362215	3.88%
TIR/Mariner (DTT)	18298	5710317	0.47%
TcMar-Tigger	4538	1618538	0.13%
TcMar-Fot1	397	309509	0.03%
TcMar-Tc1	589	327503	0.03%
TcMar-Tc2	123	20682	0.00%
Tc-Mar unclassified	12651	3434085	0.28%
TIR/CACTA (DTC)	215214	46791698	3.83%
TIR/Harbinger (DTH)	24002	5680502	0.46%
TIR/PiggyBac (DTB)	2868	1801225	0.15%
TIR/P element (DTP)	898	432278	0.04%
TIR/Merlin (DTE)	147	73034	0.01%
Helitron (DHH)	122035	31337168	2.56%
Other	16688	10344937	0.85%
TIR/Sola2	1512	598700	0.05%
Academ-1	257	196268	0.02%
CMC-Chapaev-3	8171	6172166	0.51%
CMC-EnSpm	78	113740	0.01%
Ginger-2	797	361041	0.03%
Kolobok-Hydra	279	165840	0.01%
MULE-MuDR	317	132183	0.01%
PIF-ISL2EU	359	108956	0.01%
Zator	4913	2494287	0.20%
Unclassified DNA transposons	5	1756	0.00%
Rolling-circles	3360	864483	0.07%

Unclassified:	958002	219520615	17.97%	355.0	379	109.2	HiC_scaffold_78_2500000-3000000 (952); HiC_scaffold_136_500000-1000000 (902); HiC_scaffold_98_0-500000 (862); HiC_scaffold_239_0-500000 (830); HiC_scaffold_1_1500000-2000000 (762)
Simple_repeat	519680	35126040	2.87%	192.5	183	102.2	HiC_scaffold_39_4500000-5000000 (734); HiC_scaffold_81_2500000-3000000 (696); HiC_scaffold_9_6000000-6500000 (600); HiC_scaffold_19_1000000-1500000 (576); HiC_scaffold_2_3500000-4000000 (569)
Low_complexity	65058	3752422	0.31%				
Total bases masked		623465343	51.03%	1012.8	1084	295.1	HiC_scaffold_50_5000000-5500000 (1631); HiC_scaffold_171_1500000-2000000 (1630); HiC_scaffold_98_0-500000 (1602); HiC_scaffold_26_0-500000 (1575); HiC_scaffold_52_2500000-3000000 (1561)

Supplementary Table 4 | Association between the location of repeat elements and protein-coding gene models in the draft genome (Btru.v1) of *Bulinus truncatus*

Repeat	Proximal to gene		Not proximal to gene		
	Number of elements	Total number of elements	Number of elements	Total number of elements	p-value
More frequently observed within 5000 nucleotides of a gene model					
Simple_repeat	8072	519680	17706	2213825	0
Low_complexity	1509	65058	24269	2668447	1.23E-207
Less frequently observed within 5000 nucleotides of a gene model					
DNA transposons					
TIR/hAT hobo-Activator (DTA)					
DNA/hAT	2273	365203	23505	2368302	1.49E-112
DNA/hAT-hATm	56	18688	25722	2714817	8.27E-26
DNA/hAT-Tip100	80	19235	25698	2714270	5.10E-17
DNA/hAT-Charlie	62	11989	25716	2721516	2.66E-07
DNA/hAT-Ac	37	6733	25741	2726772	0.000481201
Mutator (DTM)	1455	192469	24323	2541036	2.19E-19
CACTA (DTC)	1697	215214	24081	2518291	4.12E-15
Helitron (DHH)	937	118704	24841	2614801	1.29E-08
R/C Helitron (DHH)	14	2943	25764	2730562	0.007048496
CMC-Chapaev-3	41	8171	25737	2725334	1.16E-05
Harbinger (DTH)	169	23767	25609	2709738	0.000154479
Ginger-2	0	797	25778	2732708	0.001242884
Retrotransposons					
LTR elements:					
Gypsy	121	24758	25657	2708747	8.51E-16
LTR unclassfied	531	103018	25247	2630487	4.01E-55
Unclassified	8071	958002	17707	1775503	1.50E-36

Supplementary Table 5 | Gene accession number and assigned ortho-groups for *Bulinus truncatus* genes involved in snail-schistosome interactions

Protein group	Gene accession number	Ortho-group
Guadeloupe resistance complex (GRC)	Btru_020374-T1; Btru_020386-T1; Btru_030869-T1; Btru_045545-T1; Btru_045546-T1; Btru_055851-T1; Btru_056054-T1; Btru_056069-T1; Btru_056070-T1; Btru_056073-T1; Btru_056074-T1	OG0002541; OG0002897; OG0002909; OG0003363; OG0003364; OG0006701; OG0010349; OG0014640
Polymorphic transmembrane cluster 2 (PTC2)	Btru_039056-T1; Btru_039063-T1; Btru_071674-T1; Btru_071748-T1; Btru_071815-T1; Btru_071843-T1; Btru_072650-T1; Btru_074129-T1	OG0000822; OG0012892; OG0014932; OG0014950; OG0023563
BIRs/IAPs	Btru_000550-T1; Btru_000556-T1; Btru_000557-T1; Btru_000560-T1; Btru_000996-T1; Btru_001789-T1; Btru_001803-T1; Btru_001804-T1; Btru_001806-T1; Btru_001809-T1; Btru_001810-T1; Btru_001812-T1; Btru_003710-T1; Btru_012179-T1; Btru_020610-T1; Btru_024576-T1; Btru_024580-T1; Btru_024583-T1; Btru_024585-T1; Btru_030863-T1; Btru_033910-T1; Btru_033911-T1; Btru_033916-T1; Btru_033917-T1; Btru_033919-T1; Btru_033920-T1; Btru_033921-T1; Btru_035946-T1; Btru_035956-T1; Btru_037543-T1; Btru_039783-T1; Btru_039784-T1; Btru_042160-T1; Btru_042161-T1; Btru_042162-T1; Btru_042163-T1; Btru_042164-T1; Btru_042166-T1; Btru_042168-T1; Btru_042170-T1; Btru_042171-T1; Btru_042174-T1; Btru_042175-T1; Btru_042176-T1; Btru_042177-T1; Btru_042178-T1; Btru_043346-T1; Btru_043351-T1; Btru_043404-T1; Btru_043405-T1; Btru_043406-T1; Btru_043407-T1; Btru_043408-T1; Btru_043410-T1; Btru_043413-T1; Btru_043414-T1; Btru_043479-T1; Btru_043480-T1; Btru_043481-T1; Btru_044031-T1; Btru_052524-T1; Btru_052528-T1; Btru_052708-T1; Btru_052709-T1; Btru_052822-T1; Btru_052827-T1; Btru_052831-T1; Btru_052836-T1; Btru_052841-T1; Btru_052846-T1; Btru_052848-T1; Btru_052852-T1; Btru_060369-T1; Btru_060370-T1; Btru_061054-T1; Btru_061072-T1; Btru_061074-T1; Btru_061076-T1; Btru_061081-T1; Btru_061083-T1; Btru_061084-T1; Btru_061091-T1; Btru_061092-T1; Btru_061095-T1; Btru_061097-T1; Btru_061099-T1; Btru_061104-T1; Btru_061105-T1; Btru_061113-T1; Btru_061120-T1; Btru_061121-T1; Btru_061125-T1; Btru_061476-T1; Btru_062084-T1; Btru_067036-T1; Btru_067037-T1; Btru_068725-T1; Btru_068726-T1; Btru_068730-T1; Btru_068731-T1; Btru_068732-T1; Btru_068733-T1; Btru_068734-T1; Btru_068738-T1; Btru_068740-T1; Btru_068741-T1; Btru_068742-T1; Btru_068743-T1; Btru_068745-T1; Btru_068748-T1; Btru_068749-T1; Btru_068750-T1; Btru_068751-T1; Btru_076517-T1; Btru_077442-T1; Btru_077545-T1; Btru_077550-T1	OG0000004; OG0000090; OG0002544; OG0002572; OG0003690; OG0004008; OG0004378; OG0006951; OG0007031; OG0007817; OG0010939; OG0012308; OG0012458; OG0012477; OG0012870; OG0013103; OG0014166; OG0014202; OG0014585; OG0014586; OG0014807; OG0016072; OG0016073; OG0016421; OG0016611; OG0016673; OG0016826; OG0016827; OG0016857; OG0016858; OG0016863; OG0016864; OG0017043; OG0017044; OG0017186; OG0017187; OG0021728; OG0022053; OG0022099; OG0022100; OG0022539; OG0022542; OG0022544; OG0022965; OG0022966; OG0023392; OG0023393; OG0023394; OG0023395
Toll-/IL-1-related proteins	Btru_000319-T1; Btru_001428-T1; Btru_002393-T1; Btru_004219-T1; Btru_004220-T1; Btru_004221-T1; Btru_004223-T1; Btru_004224-T1; Btru_004225-T1; Btru_004238-T1; Btru_004239-T1; Btru_004241-T1; Btru_004242-T1; Btru_004243-T1; Btru_008082-T1; Btru_012751-T1; Btru_020061-T1; Btru_020064-T1; Btru_021443-T1; Btru_021444-T1; Btru_021445-T1; Btru_021452-T1; Btru_021453-T1; Btru_022774-T1; Btru_022776-T1; Btru_023114-T1; Btru_023122-T1; Btru_024553-T1; Btru_025254-T1; Btru_025346-T1; Btru_025608-T1; Btru_025610-T1; Btru_025624-T1; Btru_025773-T1; Btru_026044-T1; Btru_026047-T1; Btru_026050-T1; Btru_026154-T1; Btru_026395-T1; Btru_026396-T1; Btru_026398-T1; Btru_026409-T1; Btru_026410-T1; Btru_026411-T1; Btru_026412-T1; Btru_027038-T1; Btru_027277-T1; Btru_028071-T1; Btru_028125-T1; Btru_028813-T1; Btru_029258-T1; Btru_029278-T1; Btru_029280-T1; Btru_029281-T1; Btru_029304-T1; Btru_029315-T1; Btru_032582-T1; Btru_032583-T1; Btru_032756-T1; Btru_032769-T1; Btru_034462-T1; Btru_035088-T1; Btru_035092-T1; Btru_035096-T1; Btru_035167-T1; Btru_035173-T1; Btru_038470-T1; Btru_040928-T1; Btru_041033-T1; Btru_041115-T1; Btru_041307-T1; Btru_041444-T1; Btru_041447-T1; Btru_041448-T1; Btru_041449-T1; Btru_043589-T1; Btru_044972-T1; Btru_045198-T1; Btru_046428-T1; Btru_046651-T1; Btru_049418-T1; Btru_049460-T1; Btru_051973-T1; Btru_051986-T1; Btru_051989-T1; Btru_052770-T1; Btru_054267-T1; Btru_054272-T1; Btru_057056-T1; Btru_058263-T1; Btru_058264-T1; Btru_058265-T1; Btru_062423-T1; Btru_062427-T1; Btru_062914-T1; Btru_062917-T1; Btru_062937-T1; Btru_063360-T1; Btru_063775-T1; Btru_063777-T1; Btru_063782-T1; Btru_064091-T1; Btru_064092-T1; Btru_064095-T1; Btru_064096-T1; Btru_064097-T1; Btru_064098-T1; Btru_064099-T1; Btru_064100-T1; Btru_066980-T1; Btru_066981-T1; Btru_067446-T1; Btru_067447-T1; Btru_067448-T1; Btru_067736-T1; Btru_068454-T1; Btru_070351-T1; Btru_070396-T1; Btru_070397-T1; Btru_070423-T1; Btru_070552-T1; Btru_071847-T1; Btru_076001-T1; Btru_076523-T1	OG0000039; OG0000371; OG0000375; OG0000380; OG0000399; OG0000449; OG0000778; OG0000914; OG0001197; OG0001465; OG0001501; OG0001578; OG0001875; OG0001894; OG0002069; OG0002261; OG0002932; OG0003092; OG0003346; OG0003680; OG0003885; OG0003980; OG0004036; OG0004345; OG0004797; OG0006724; OG0006975; OG0008394; OG0009557; OG0009649; OG0009858; OG0010244; OG0010317; OG0010844; OG0010956; OG0011566; OG0011869; OG0012506; OG0012824; OG0013822; OG0014933; OG0016985; OG0021556; OG0021690; OG0021691; OG0022183; OG0022196; OG0023098; OG0023497
Cathepsins	Btru_012554-T1; Btru_012560-T1; Btru_012579-T1; Btru_012580-T1; Btru_012588-T1; Btru_012589-T1; Btru_022942-T1; Btru_045113-T1; Btru_045118-T1; Btru_050025-T1; Btru_054689-T1; Btru_054690-T1; Btru_056014-T1; Btru_056775-T1; Btru_058393-T1; Btru_061634-T1; Btru_063756-T1; Btru_063757-T1; Btru_063758-T1; Btru_064987-T1; Btru_077978-T1	OG0000133; OG0000596; OG0001616; OG0006192; OG0006645; OG0012606; OG0020952

Chitinases	Btru_003083-T1; Btru_013557-T1; Btru_013559-T1; Btru_020862-T1; Btru_021015-T1; Btru_021016-T1; Btru_021215-T1; Btru_021586-T1; Btru_023115-T1; Btru_023131-T1; Btru_023560-T1; Btru_023562-T1; Btru_023833-T1; Btru_023834-T1; Btru_025249-T1; Btru_025266-T1; Btru_025271-T1; Btru_025272-T1; Btru_025275-T1; Btru_025276-T1; Btru_025621-T1; Btru_030528-T1; Btru_030666-T1; Btru_030673-T1; Btru_031327-T1; Btru_033484-T1; Btru_033838-T1; Btru_033840-T1; Btru_034762-T1; Btru_035548-T1; Btru_035550-T1; Btru_035552-T1; Btru_035553-T1; Btru_038813-T1; Btru_039952-T1; Btru_039956-T1; Btru_041059-T1; Btru_041062-T1; Btru_041071-T1; Btru_041087-T1; Btru_041747-T1; Btru_041748-T1; Btru_041813-T1; Btru_041826-T1; Btru_041834-T1; Btru_042810-T1; Btru_042837-T1; Btru_042848-T1; Btru_042862-T1; Btru_043200-T1; Btru_043201-T1; Btru_043208-T1; Btru_043209-T1; Btru_043853-T1; Btru_043863-T1; Btru_047181-T1; Btru_047192-T1; Btru_047194-T1; Btru_047195-T1; Btru_049893-T1; Btru_050039-T1; Btru_050043-T1; Btru_050044-T1; Btru_050504-T1; Btru_051405-T1; Btru_053451-T1; Btru_053452-T1; Btru_053454-T1; Btru_053455-T1; Btru_053800-T1; Btru_055795-T1; Btru_059014-T1; Btru_059015-T1; Btru_059333-T1; Btru_059340-T1; Btru_059341-T1; Btru_059344-T1; Btru_059437-T1; Btru_059451-T1; Btru_061747-T1; Btru_064158-T1; Btru_065356-T1; Btru_066843-T1; Btru_066844-T1; Btru_067551-T1; Btru_067557-T1; Btru_067558-T1; Btru_067561-T1; Btru_068845-T1; Btru_068846-T1; Btru_069743-T1; Btru_070098-T1; Btru_070664-T1; Btru_070684-T1; Btru_070735-T1; Btru_072445-T1; Btru_072451-T1; Btru_072452-T1; Btru_072453-T1; Btru_072601-T1; Btru_073457-T1; Btru_074476-T1; Btru_074562-T1	OG0000066; OG0000085; OG0000161; OG0000246; OG0000349; OG0001157; OG0001438; OG0001507; OG0010229; OG0011097; OG0012060; OG0012914; OG0013086; OG0014414; OG0016765; OG0017110; OG0021285; OG0022423
Calmodulins	Btru_002791-T1; Btru_002810-T1; Btru_002812-T1; Btru_006472-T1; Btru_006792-T1; Btru_007456-T1; Btru_009705-T1; Btru_009714-T1; Btru_009743-T1; Btru_012064-T1; Btru_012066-T1; Btru_012088-T1; Btru_012090-T1; Btru_014179-T1; Btru_017949-T1; Btru_027601-T1; Btru_027602-T1; Btru_027604-T1; Btru_027605-T1; Btru_027645-T1; Btru_030589-T1; Btru_035684-T1; Btru_037768-T1; Btru_037779-T1; Btru_038975-T1; Btru_041670-T1; Btru_041671-T1; Btru_042139-T1; Btru_044011-T1; Btru_044017-T1; Btru_048713-T1; Btru_053654-T1; Btru_053664-T1; Btru_056554-T1; Btru_056555-T1; Btru_056557-T1; Btru_056571-T1; Btru_056573-T1; Btru_064116-T1; Btru_069807-T1; Btru_070968-T1; Btru_075886-T1	OG0000309; OG0000320; OG0000714; OG0002453; OG0004066; OG0004258; OG0004357; OG0006198; OG0006292; OG0006489; OG0006972; OG0008499; OG0009340; OG0010873; OG0011491; OG0012074; OG0012176; OG0012911; OG0015773; OG0016269; OG0016544; OG0016545; OG0020656; OG0020657; OG0020938; OG0021013; OG0022579; OG0023454
Lectins	Btru_004755-T1; Btru_004758-T1; Btru_004766-T1; Btru_005062-T1; Btru_005065-T1; Btru_005646-T1; Btru_006267-T1; Btru_008140-T1; Btru_009287-T1; Btru_009304-T1; Btru_012490-T1; Btru_012497-T1; Btru_012523-T1; Btru_012534-T1; Btru_012537-T1; Btru_015108-T1; Btru_020400-T1; Btru_020403-T1; Btru_021298-T1; Btru_024590-T1; Btru_024591-T1; Btru_027826-T1; Btru_029650-T1; Btru_030062-T1; Btru_030123-T1; Btru_030128-T1; Btru_030129-T1; Btru_030693-T1; Btru_031469-T1; Btru_031916-T1; Btru_032911-T1; Btru_035110-T1; Btru_035116-T1; Btru_035117-T1; Btru_037259-T1; Btru_038635-T1; Btru_038649-T1; Btru_042928-T1; Btru_045040-T1; Btru_045100-T1; Btru_045525-T1; Btru_045526-T1; Btru_045533-T1; Btru_045534-T1; Btru_046814-T1; Btru_046822-T1; Btru_046827-T1; Btru_046828-T1; Btru_047253-T1; Btru_047257-T1; Btru_047408-T1; Btru_047410-T1; Btru_047777-T1; Btru_049345-T1; Btru_049432-T1; Btru_049448-T1; Btru_049902-T1; Btru_049947-T1; Btru_050005-T1; Btru_050461-T1; Btru_051552-T1; Btru_054231-T1; Btru_054905-T1; Btru_055548-T1; Btru_055611-T1; Btru_055612-T1; Btru_055723-T1; Btru_057243-T1; Btru_057502-T1; Btru_058565-T1; Btru_061222-T1; Btru_061224-T1; Btru_061226-T1; Btru_061232-T1; Btru_061233-T1; Btru_061236-T1; Btru_061239-T1; Btru_062643-T1; Btru_062898-T1; Btru_063025-T1; Btru_063033-T1; Btru_063047-T1; Btru_063050-T1; Btru_063056-T1; Btru_063080-T1; Btru_063085-T1; Btru_063089-T1; Btru_063090-T1; Btru_063092-T1; Btru_063099-T1; Btru_063404-T1; Btru_063598-T1; Btru_065141-T1; Btru_065474-T1; Btru_065478-T1; Btru_065479-T1; Btru_067597-T1; Btru_070385-T1; Btru_071573-T1; Btru_071897-T1; Btru_073089-T1	OG0000022; OG0000032; OG0000075; OG0000081; OG0000234; OG0000395; OG0000661; OG0000683; OG0001047; OG0001190; OG0001485; OG0002074; OG0002715; OG0002882; OG0002919; OG0004266; OG0004320; OG0006282; OG0006971; OG0010730; OG0012158; OG0012265; OG0013043; OG0013104; OG0013123; OG0013155; OG0014600; OG0014664; OG0014751; OG0014955; OG0017361; OG0022277; OG0022306
Fibrinogen-related proteins[b]	Btru_002050-T1; Btru_002355-T1; Btru_002357-T1; Btru_002359-T1; Btru_002414-T1; Btru_002417-T1; Btru_002418-T1; Btru_002419-T1; Btru_002421-T1; Btru_002562-T1; Btru_002563-T1; Btru_002565-T1; Btru_002567-T1; Btru_002568-T1; Btru_002569-T1; Btru_002571-T1; Btru_002573-T1; Btru_002592-T1; Btru_002595-T1; Btru_002844-T1; Btru_005787-T1; Btru_007887-T1; Btru_008107-T1; Btru_011637-T1; Btru_014330-T1; Btru_015335-T1; Btru_017645-T1; Btru_017646-T1; Btru_017649-T1; Btru_017659-T1; Btru_017665-T1; Btru_020607-T1; Btru_020880-T1; Btru_020886-T1; Btru_020891-T1; Btru_026655-T1; Btru_028570-T1; Btru_028592-T1; Btru_033525-T1; Btru_033549-T1; Btru_033660-T1; Btru_033682-T1; Btru_033684-T1; Btru_033719-T1; Btru_033722-T1; Btru_033726-T1; Btru_036502-T1; Btru_037327-T1; Btru_037329-T1; Btru_037330-T1; Btru_037331-T1; Btru_037335-T1; Btru_038382-T1; Btru_042805-T1; Btru_042813-T1; Btru_044410-T1; Btru_044417-T1; Btru_045437-T1; Btru_045438-T1; Btru_045439-T1; Btru_045443-T1; Btru_045444-T1; Btru_045445-T1; Btru_045447-T1; Btru_045456-T1; Btru_045457-T1; Btru_045459-T1; Btru_046358-T1; Btru_046364-T1; Btru_048070-T1; Btru_048079-T1; Btru_048110-T1; Btru_048335-T1; Btru_048454-T1; Btru_048570-T1; Btru_048571-T1; Btru_048572-T1; Btru_048597-T1; Btru_048813-T1; Btru_048819-T1; Btru_050088-T1; Btru_051619-T1; Btru_051642-T1; Btru_054121-T1; Btru_054126-T1; Btru_054688-T1; Btru_056151-T1; Btru_056154-T1; Btru_056155-T1; Btru_058726-T1; Btru_059143-T1; Btru_059171-T1; Btru_059358-T1; Btru_059359-T1; Btru_059373-T1; Btru_059383-T1; Btru_059384-T1; Btru_059385-T1; Btru_059387-T1; Btru_059388-T1; Btru_060419-T1; Btru_060420-T1; Btru_060567-T1; Btru_063385-T1; Btru_063402-T1; Btru_063403-T1; Btru_063406-T1; Btru_063407-T1; Btru_063413-T1; Btru_063414-T1; Btru_063417-T1; Btru_063419-T1; Btru_063424-T1; Btru_065328-T1; Btru_065330-T1; Btru_065331-T1; Btru_066440-T1; Btru_066610-T1; Btru_066611-T1; Btru_067843-T1; Btru_067844-T1; Btru_067847-T1; Btru_067904-T1; Btru_067908-T1; Btru_069814-T1; Btru_075127-T1; Btru_075340-T1; Btru_075352-T1; Btru_077286-T1; Btru_077287-T1	OG0000016; OG0000103; OG0000581; OG0000963; OG0001504; OG0003488; OG0004040; OG0004042; OG0004264; OG0004492; OG0006406; OG0006460; OG0006657; OG0009439; OG0009714; OG0010992; OG0012970; OG0014832; OG0014999; OG0016321; OG0016669; OG0016920; OG0020653; OG0020745; OG0020812; OG0022426; OG0022925; OG0023258; OG0023928

Supplementary Table 6 | Classification of fibrinogen-related lectins in the proteomes of *Bulinus truncatus* and *Biomphalaria glabrata*.

		Homology to Pfam PF00147 fibrinogen beta and gamma chains, C-terminal globular domain					
Gene accession	Length	E-value	Score	Number of FreD domains	Published classification a	Class	*B. truncatus* FReD-like orthogroup
BGLB000019-PA	601	6.80E-63	198.5	1	2-IgSF_FREP	C	
BGLB000021-PB	614	1.70E-64	203.7	1	2-IgSF_FREP	C	
BGLB000074-PB	616	1.10E-56	178.2	1	2-IgSF_FREP	C	
BGLB000096-PA	634	2.20E-60	190.3	1	2-IgSF_FREP	C	
BGLB000100-PA	640	3.20E-61	193.1	3	2-IgSF_FREP	C	
BGLB000116-PB	811	7.20E-77	244.2	3	2-IgSF_FREP	C	
BGLB000131-PA	756	1.80E-40	125.2	2	2-IgSF_FREP	C	
BGLB000133-PA	634	1.90E-63	200.3	1	2-IgSF_FREP	C	
BGLB000140-PA	633	3.90E-60	189.5	1	2-IgSF_FREP	C	
BGLB000141-PA	405	8.20E-36	110	1	1-IgSF_FREP	B	
BGLB000152-PB	400	2.10E-57	180.6	1	1-IgSF_FREP	B	
BGLB000177-PB	255	7.60E-59	185.3	1	sFReD	A with helix	
BGLB000178-PA	223	7.80E-64	201.6	1	sFReD	A with helix	OG0000016
BGLB000179-PA	554	1.30E-62	197.6	1	1-IgSF_FREP	B	
BGLB000204-PB	412	3.00E-62	196.4	1	2-IgSF_FREP	C	
BGLB002450-PB	222	2.50E-40	124.7	1	sFReD	A	
BGLB002494-PB	212	5.70E-59	185.7	1	sFReD	A	
BGLB004529-PB	622	1.10E-55	174.9	1	2-IgSF_FREP	C	
BGLB004530-PB	634	3.10E-62	196.3	1	2-IgSF_FREP	C	
BGLB005196-PB	389	2.90E-58	183.4	1	sFReD	B	
BGLB005224-PB	685	4.70E-61	192.5	1	2-IgSF_FREP	C	
BGLB006034-PB	536	2.20E-12	33.3	1	2-IgSF_FREP	C	
BGLB007076-PB	715	8.70E-63	198.2	1	2-IgSF_FREP	C	
BGLB007576-PB	571	2.60E-55	173.7	2	2-IgSF_FREP	C	

BGLB010948-PB	638	8.10E-13	34.8	1	2-IgSF_FREP	C	
BGLB011626-PB	722	6.40E-66	208.4	1	2-IgSF_FREP	C	
BGLB011627-PB	741	5.20E-65	205.4	1	2-IgSF_FREP	C	
BGLB012039-PB	172	2.30E-33	102	1	sFReD	A	
BGLB012226-PB	196	1.00E-54	171.7	1	sFReD	A	
BGLB012382-PB	366	3.30E-59	186.4	2	sFReD	A with helix	OG0006406
BGLB014428-PB	381	1.60E-60	190.8	1	1-IgSF_FREP	B	
BGLB016307-PA	308	4.00E-57	179.6	1	sFReD	A	
BGLB016436-PA	78	1.20E-16	47.3	1	sFReD	A	
BGLB016934-PA	587	2.80E-58	183.4	1	2-IgSF_FREP	C	
BGLB016935-PA	806	2.80E-61	193.2	1	2-IgSF_FREP	C	
BGLB017041-PA	219	1.20E-50	158.4	1	sFReD	A	
BGLB017605-PA	235	4.50E-58	182.8	1	sFReD	A with helix	
BGLB017688-PA	503	4.80E-62	195.7	1	2-IgSF_FREP	C	
BGLB017893-PA	622	2.70E-59	186.8	1	2-IgSF_FREP	C	
BGLB018336-PA	217	3.00E-62	196.4	1	sFReD	A	
BGLB020351-PA	296	2.20E-56	177.2	1	sFReD	A with beta and helix	
BGLB020516-PA	273	9.30E-58	181.7	1	2-IgSF_FREP	A with helix	
BGLB021912-PA	253	2.00E-19	56.4	1	sFReD	A with beta and helix	OG0004042
BGLB022646-PA	555	6.60E-64	201.8	1	1-IgSF_FREP	B	
BGLB022867-PA	260	4.90E-19	55.1	1	sFReD	A with beta and helix	
BGLB023857-PA	528	2.00E-63	200.2	1	1-IgSF_FREP	B	
BGLB025144-PA	290	3.20E-66	209.4	1	2-IgSF_FREP	A with helix	
BGLB025334-PA	473	2.20E-62	196.9	1	1-IgSF_FREP	B	
BGLB025627-PA	136	9.30E-37	113	1	sFReD	A	
BGLB025643-PA	281	1.80E-61	193.9	1	sFReD	A	OG0000016
BGLB028385-PA	235	3.30E-69	219.2	1	sFReD	A	OG0000016
BGLB028617-PA	366	5.90E-64	202	1	1-IgSF_FREP	B	
BGLB029167-PA	434	6.40E-61	192.1	3	1-IgSF_FREP	B	
BGLB029288-PA	112	1.10E-13	37.6	1	sFReD	A	

BGLB030478-PA	106	1.60E-24	73.1	1	sFReD	A	
BGLB030830-PA	96	9.70E-14	37.8	1	sFReD	A	
BGLB031381-PA	222	1.70E-61	193.9	1	sFReD	A with helix	OG0000016
BGLB031596-PA	139	1.60E-29	89.4	1	sFReD	A	
BGLB032905-PA	281	7.80E-53	165.6	1	2-IgSF_FREP	A with helix	
BGLB034525-PA	86	3.00E-23	68.9	1	sFReD	A	
BGLB036251-PA	222	2.00E-62	197	1	sFReD	A	OG0000016
BGLB037621-PA	439	6.90E-19	54.6	2	sFReD	A with helix	
BGLB038117-PA	119	2.50E-22	65.9	1	sFReD	A	
BGLB038186-PA	200	1.60E-54	171.1	1	sFReD	A	
BGLB038205-PA	572	4.30E-48	150.1	2	1-IgSF_FREP	B	
BGLB038723-PA	212	3.00E-57	180	1	sFReD	A	
BGLB039918-PA	374	2.50E-20	59.3	1	sFReD	A with beta and helix	OG0004042
BGLB040034-PA	153	5.40E-58	182.5	1	sFReD	A	OG0000016
BGLB040226-PA	331	1.70E-64	203.7	1	2-IgSF_FREP	A with helix	
BGLB040228-PA	407	3.10E-65	206.2	1	1-IgSF_FREP	B	
BGLB040339-PB	395	1.30E-71	227	1	sFReD	A with helix	
BGLB040437-PA	499	1.00E-17	50.8	3	FReM	D	
Btru_002050-T1	278	1.30E-47	148.5	1		A with helix	OG0000103
Btru_002355-T1	184	1.20E-47	148.7	1		A	OG0000103
Btru_002357-T1	423	6.00E-19	54.8	1		A-like	OG0000103
Btru_002359-T1	615	1.40E-47	148.5	1		A	OG0000103
Btru_002414-T1	178	2.20E-43	134.7	1		A-like	OG0000103
Btru_002417-T1	216	5.00E-53	166.3	1		A with helix	OG0000103
Btru_002418-T1	214	3.40E-52	163.5	1		A	OG0000103
Btru_002419-T1	109	7.80E-25	74.1	1		A-like	OG0000103
Btru_002421-T1	289	6.10E-56	175.8	1		A	OG0000103
Btru_002562-T1	221	3.80E-52	163.4	1		A	OG0000103
Btru_002563-T1	225	3.50E-56	176.6	1		A	OG0000103
Btru_002565-T1	186	6.10E-47	146.4	1		A-like	
Btru_002567-T1	223	1.90E-53	167.7	1		A	OG0000103

Btru_002568-T1	222	9.50E-53	165.3	1	A	OG0000103
Btru_002569-T1	228	7.50E-57	178.7	1	A	OG0000103
Btru_002571-T1	229	3.80E-55	173.2	1	A	OG0000103
Btru_002573-T1	306	9.70E-57	178.4	1	A with helix	OG0000103
Btru_002592-T1	156	1.80E-30	92.5	1	A-like	
Btru_002595-T1	145	3.70E-40	124.2	1	A-like	
Btru_002844-T1	209	3.70E-54	169.9	1	A	OG0000103
Btru_005787-T1	389	1.20E-21	63.6	1	A with beta and helix	
Btru_007887-T1	837	2.10E-23	69.4	1	C	
Btru_008107-T1	322	5.20E-57	179.3	1	A with beta and helix	OG0001504
Btru_011637-T1	260	1.20E-74	236.9	1	A with beta and helix	
Btru_014330-T1	241	4.00E-55	173.1	1	A with helix	OG0001504
Btru_015335-T1	247	1.60E-09	24	1	A-like	
Btru_017645-T1	629	2.10E-57	180.6	1	A-like	OG0000016
Btru_017646-T1	276	1.30E-61	194.4	1	A with beta and helix	OG0000016
Btru_017649-T1	149	2.20E-57	180.5	1	A-like	OG0000016
Btru_017659-T1	209	5.50E-55	172.7	1	A	OG0000016
Btru_017665-T1	221	6.80E-63	198.5	1	A	OG0000016
Btru_020607-T1	224	1.10E-52	165.1	1	A	OG0000103
Btru_020880-T1	224	1.10E-53	168.3	1	A	OG0000103
Btru_020886-T1	224	1.80E-54	171	1	A	OG0000103
Btru_020891-T1	223	3.90E-53	166.6	1	A	OG0000103
Btru_026655-T1	352	2.60E-62	196.6	1	A with beta and helix	OG0000016
Btru_028592-T1	405	1.50E-35	109.1	1	B	
Btru_033525-T1	217	5.40E-47	146.5	1	A	
Btru_033549-T1	213	3.30E-41	127.6	1	A	
Btru_033660-T1	220	1.80E-62	197.1	1	A	OG0000016
Btru_033682-T1	483	1.60E-34	105.7	1	A-like	OG0000016

Btru_033684-T1	225	1.50E-63	200.7	1	A	OG0000016
Btru_033719-T1	423	1.40E-57	181.1	2	E	OG0000016
Btru_033722-T1	108	1.60E-30	92.6	1	A-like	OG0000016
Btru_033726-T1	225	2.90E-62	196.5	1	A	OG0000016
Btru_036502-T1	211	2.30E-22	66	2	A-like	
Btru_037327-T1	173	8.80E-45	139.3	1	A	OG0001504
Btru_037329-T1	269	4.70E-54	169.6	1	A with helix	OG0001504
Btru_037330-T1	220	5.30E-53	166.2	1	A	OG0001504
Btru_037331-T1	221	1.60E-53	167.9	1	A	OG0001504
Btru_037335-T1	335	4.70E-47	146.7	3	A-like	OG0001504
Btru_038382-T1	237	1.50E-68	217	1	A	OG0000016
Btru_042805-T1	356	1.50E-52	164.7	2	A with helix	OG0000581
Btru_042813-T1	141	1.60E-29	89.4	1	A-like	OG0000581
Btru_045437-T1	261	4.60E-64	202.3	2	A with helix	OG0000016
Btru_045438-T1	235	5.20E-63	198.9	1	A with beta and helix	OG0000016
Btru_045439-T1	222	7.90E-54	168.9	1	A with helix	OG0000016
Btru_045443-T1	222	7.20E-56	175.5	1	A with helix	OG0000016
Btru_045444-T1	233	1.30E-63	200.9	1	A with beta and helix	OG0000016
Btru_045445-T1	206	1.70E-40	125.3	2	A-like	OG0000016
Btru_045447-T1	232	1.30E-64	204.2	1	A	OG0000016
Btru_045456-T1	240	4.60E-64	202.3	1	A with helix	OG0000016
Btru_045457-T1	169	4.20E-43	133.8	1	A	OG0000016
Btru_045459-T1	123	2.60E-19	56	1	A-like	OG0000016
Btru_046358-T1	226	1.80E-31	95.8	1	A-like	
Btru_046364-T1	287	1.20E-51	161.7	1	A with helix	
Btru_048070-T1	242	5.30E-51	159.6	1	A	OG0010992
Btru_048079-T1	212	2.90E-50	157.2	1	A	OG0010992
Btru_048110-T1	656	5.50E-46	143.2	1	F	OG0010992
Btru_048335-T1	439	6.50E-70	221.5	2	A with helix	
Btru_048570-T1	249	5.30E-24	71.3	1	A-like	OG0000103

Btru_048571-T1	240	3.20E-53	166.9	1	A	OG0000103
Btru_048572-T1	274	2.60E-54	170.4	1	A with helix	OG0000103
Btru_048597-T1	521	1.60E-96	308.5	2	A-like	OG0000103
Btru_048813-T1	275	3.50E-42	130.8	1	A with helix	
Btru_048819-T1	228	4.80E-49	153.2	1	A with helix	
Btru_050088-T1	569	3.30E-48	150.5	2	A with helix	
Btru_051642-T1	381	6.70E-49	152.8	2	A with helix	OG0006406
Btru_054121-T1	387	6.00E-23	67.9	1	A with beta and helix	OG0004042
Btru_054126-T1	653	2.10E-23	69.4	1	A with beta and helix	OG0004042
Btru_054688-T1	288	7.60E-24	70.8	1	A with beta and helix	OG0004042
Btru_056151-T1	219	8.60E-33	100.1	1	A with helix	OG0000103
Btru_056154-T1	224	6.30E-54	169.2	1	A	OG0000103
Btru_056155-T1	217	2.70E-50	157.3	1	A	OG0000103
Btru_058726-T1	218	4.30E-56	176.3	1	A	OG0001504
Btru_059143-T1	214	2.00E-54	170.8	1	A	OG0000581
Btru_059171-T1	194	1.00E-54	171.8	1	A	OG0000581
Btru_059358-T1	210	1.90E-50	157.8	1	A	OG0000016
Btru_059359-T1	225	1.30E-61	194.3	1	A	OG0000016
Btru_059373-T1	223	2.20E-60	190.3	1	A	OG0000016
Btru_059383-T1	221	7.30E-41	126.5	1	A-like	OG0000016
Btru_059384-T1	253	9.30E-62	194.8	1	A	OG0000016
Btru_059385-T1	217	1.70E-54	171	1	A	OG0000016
Btru_059387-T1	188	6.60E-43	133.1	2	A-like	OG0000016
Btru_059388-T1	220	4.50E-61	192.5	1	A	OG0000016
Btru_060419-T1	270	1.60E-10	27.3	1	A-like	
Btru_060420-T1	604	2.90E-26	78.7	3	A	
Btru_060567-T1	228	1.90E-50	157.8	1	A	OG0000103
Btru_063385-T1	408	9.80E-25	73.7	1	A-like	OG0014832
Btru_063402-T1	213	7.90E-59	185.2	1	A	OG0000581
Btru_063403-T1	184	3.90E-23	68.5	1	A-like	OG0014832

Btru_063406-T1	303	8.90E-52	162.2	1	A with helix	OG0000581
Btru_063407-T1	213	1.40E-57	181.1	1	A	OG0000581
Btru_063413-T1	213	8.10E-48	149.2	1	A	OG0000581
Btru_063414-T1	233	7.00E-51	159.2	1	A with helix	OG0000581
Btru_063417-T1	217	4.70E-50	156.5	1	A	OG0000581
Btru_063419-T1	217	2.90E-50	157.2	1	A	OG0000581
Btru_063424-T1	213	1.70E-57	180.9	1	A	OG0000581
Btru_065328-T1	229	1.70E-49	154.7	1	A with helix	OG0000581
Btru_065330-T1	221	4.70E-52	163.1	1	A	OG0000581
Btru_065331-T1	215	2.00E-38	118.5	1	A-like	OG0000581
Btru_066610-T1	346	2.90E-31	95.1	1	A-like	OG0001504
Btru_066611-T1	197	2.30E-41	128.1	1	A	OG0001504
Btru_067843-T1	185	3.30E-46	143.9	1	A	OG0000016
Btru_067844-T1	223	1.40E-62	197.4	1	A	OG0000016
Btru_067847-T1	309	2.50E-59	186.8	1	A with beta and helix	OG0000016
Btru_067904-T1	316	4.50E-62	195.8	1	A with beta and helix	OG0000016
Btru_067908-T1	314	1.90E-60	190.5	1	A with beta and helix	OG0000016
Btru_069814-T1	178	5.10E-29	87.7	2	A-like	OG0000016
Btru_075127-T1	224	7.60E-53	165.6	1	A	OG0000103
Btru_075340-T1	271	1.10E-56	178.2	2	A with helix	OG0000103
Btru_075352-T1	327	7.40E-57	178.8	1	A with beta and helix	OG0000103
Btru_077286-T1	119	4.70E-25	74.8	1	A-like	
Btru_077287-T1	157	2.50E-25	75.7	1	A-like	

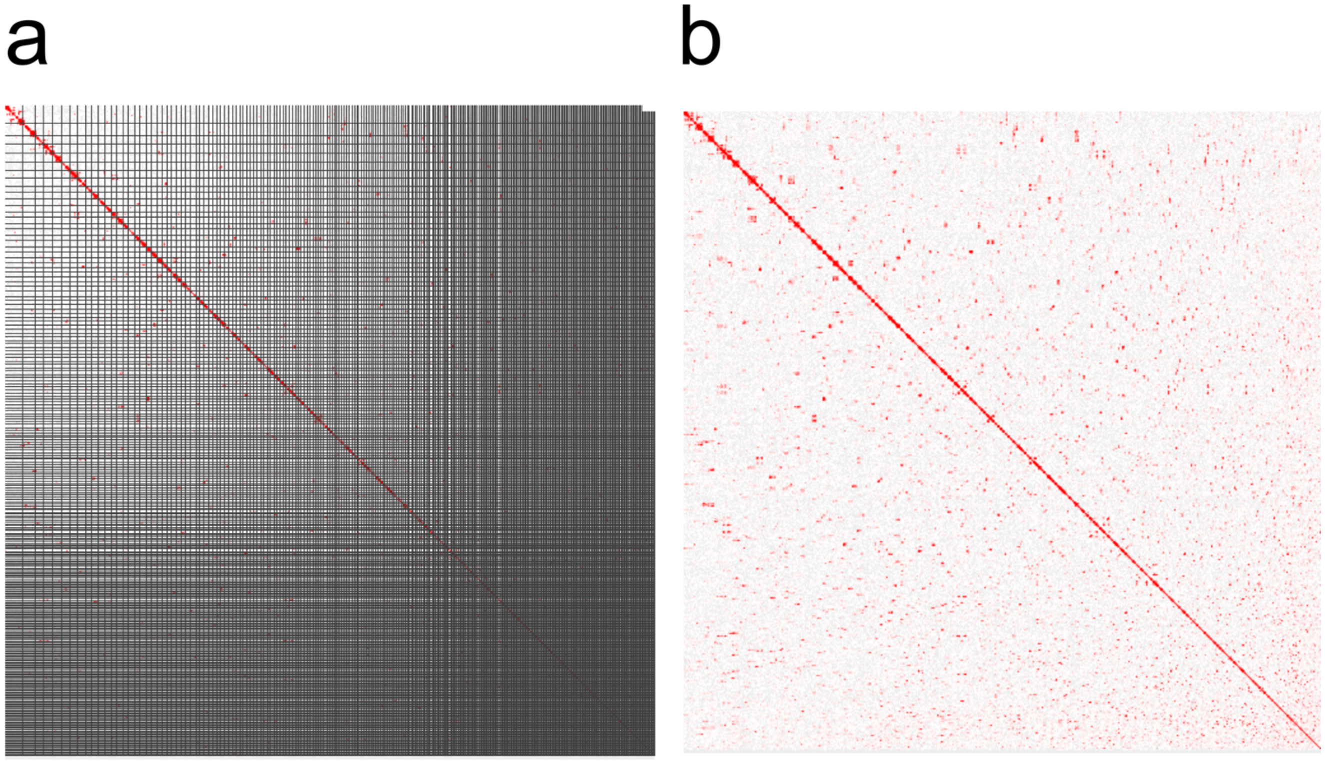

Supplementary Fig. 1 Spatial clustering of Hi-C data. Matrix of the spatial clustering of Hi-C sequence reads to the 553 scaffolds (a) representing the draft genome (Btru.v1) of *Bulinus truncatus*; clustering without scaffold delineations (b).

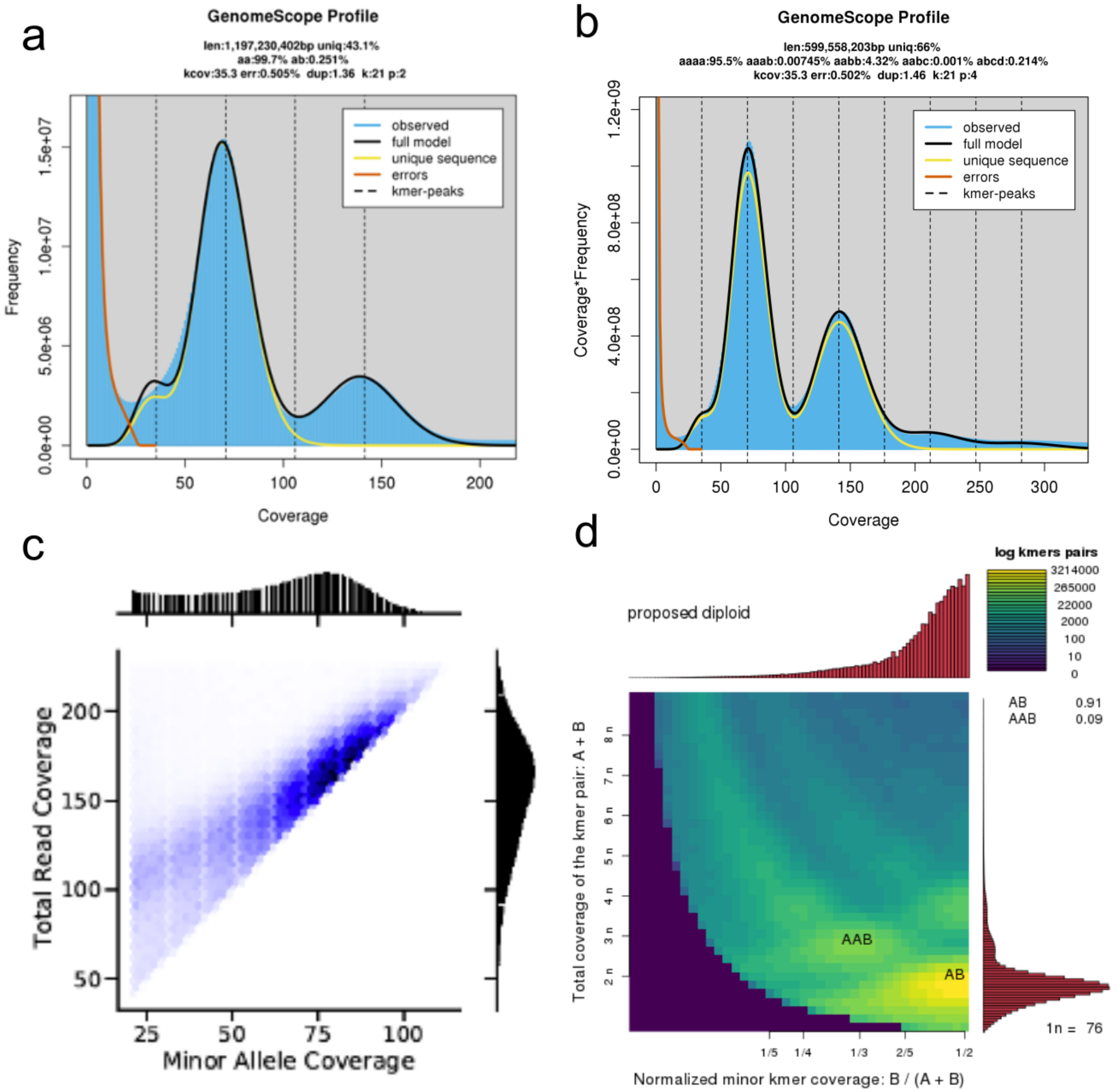

Supplementary Fig. 2 Ploidy assessment. Estimates of ploidy for the reference laboratory strain of *Bulinus truncatus* (BRI strain) using a short-read (~500 bp) data obtained using Illumina sequencing. Panel **a**: GenomeScope2 21-mer profile, assuming a diploid genome model. Panel **b**: GenomeScope2 21-mer profiles, assuming a tetraploid genome model. Panel **c**: Estimated association between total read coverage and minor allele frequency using reads that mapped to the assembled genome of *Bu. truncatus*. Panel **d**: Log-transformed SmudgePlot profile of 21-mer paired coverage indicating diploidy among *Bu. truncatus* short-read (Illumina) DNA data. Abbreviations: length (len); base pairs (bp); unique 21-mers (uniq); estimated 21-mer coverage (kcov); heterozygosity (err); k-mer selected (k); ploidy model (p).

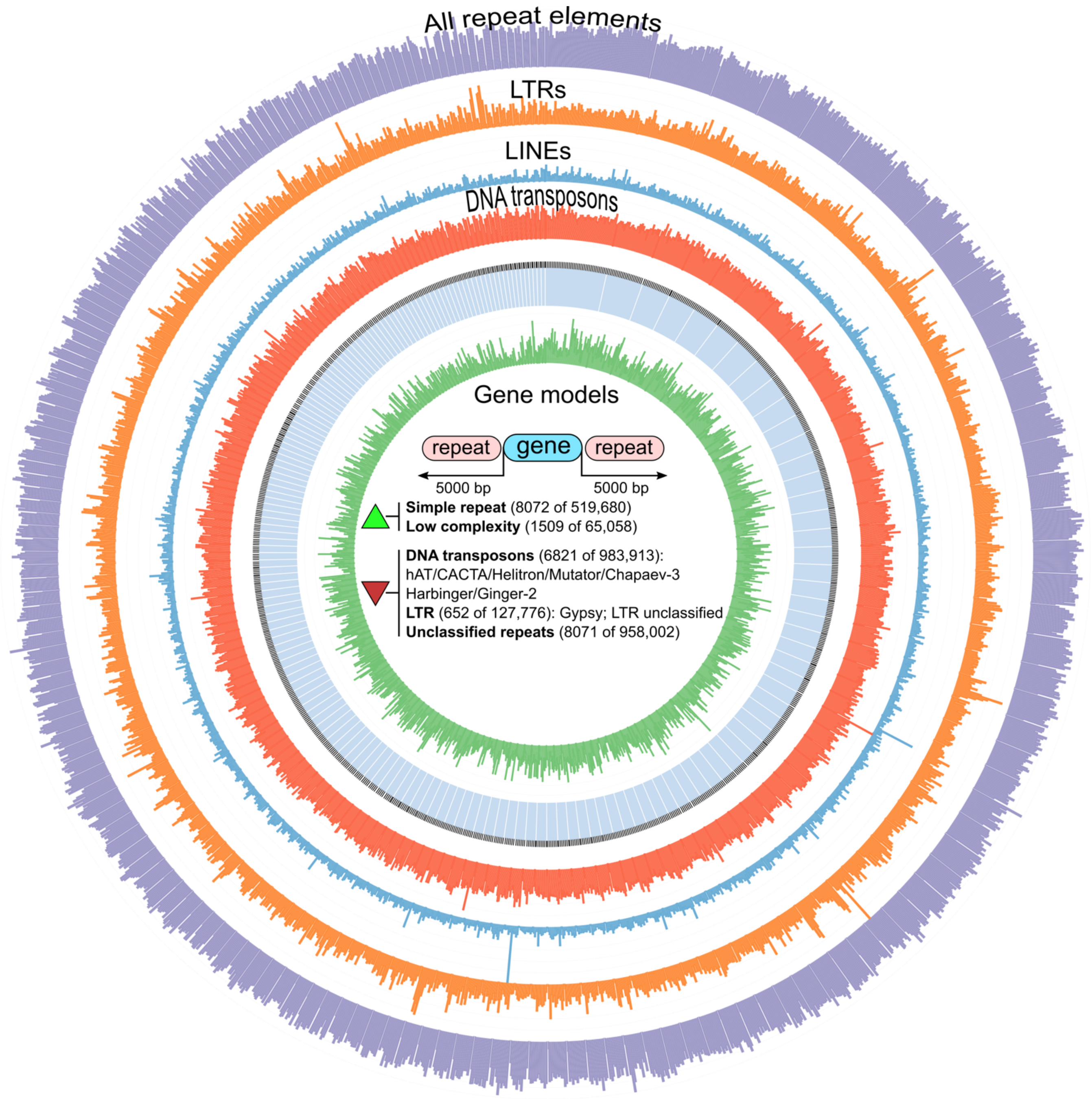

Supplementary Fig. 3 Distribution of repeat elements in the *Bulinus truncatus* genome. Genome scaffolds of > 2×10^6 nucleotides in length (~81% of the nuclear genome) are shown (light blue) and tick marks represent each 1×10^6 nucleotides. The inner histogram (green) displays the number of gene models per 5×10^5 of non-overlapping nucleotides. Outer histograms represent the distribution of repeat elements in 5×10^5 of non-overlapping windows, including all repeat elements (purple), long terminal repeat retrotransposons (LTRs; orange), long interspersed nuclear elements (LINEs; blue) and DNA transposons (red). Elements that are more (green triangle) or less (red triangle) likely to be located within 5000 nucleotides of a predicted gene model are indicated.

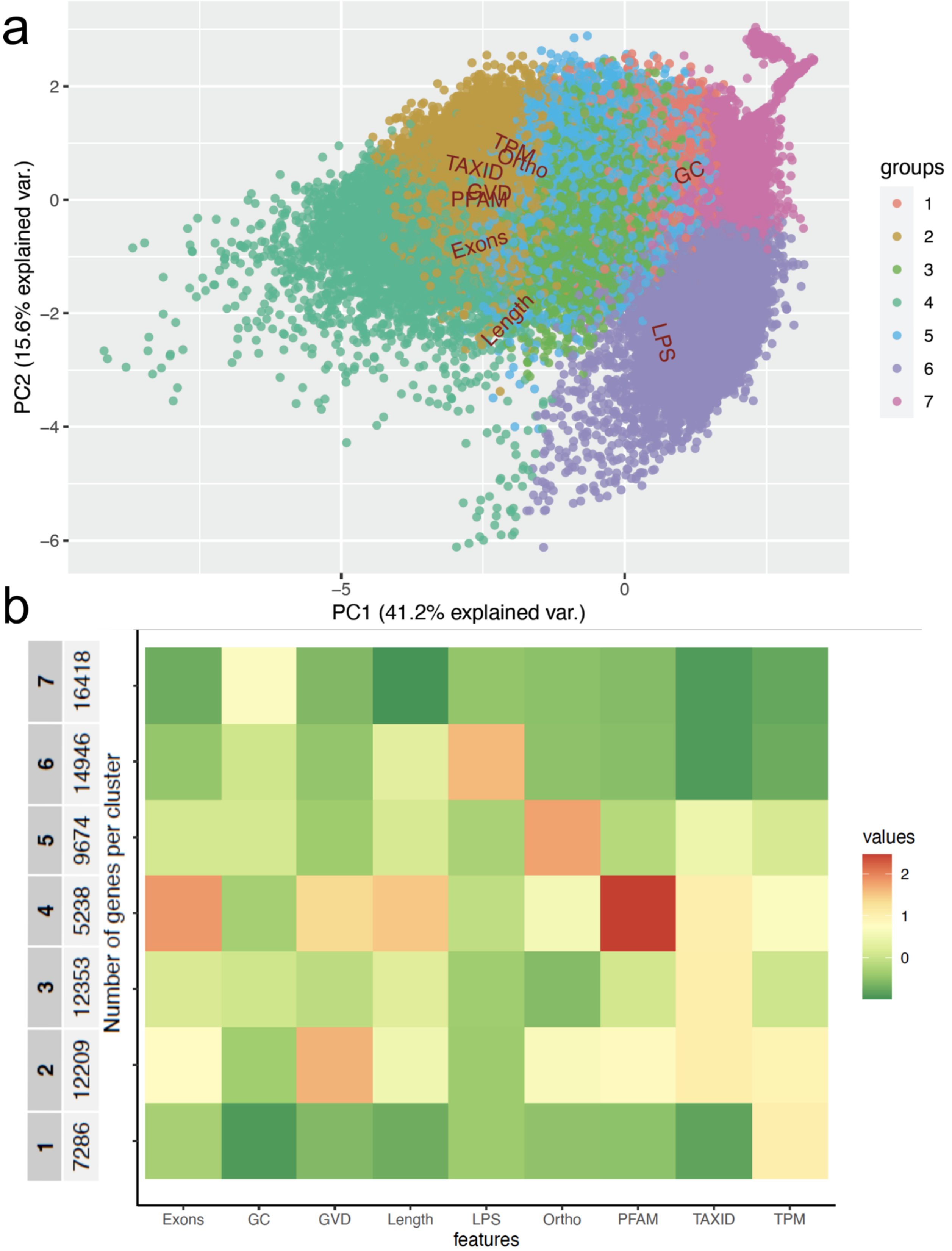

Supplementary Fig. 4 Selection of gene models. Selection of final gene models for *Bulinus truncatus*. Features curated included: number of exons (Exons); GC content (GC); GeneValidator score (GVD); mRNA length (Length); proportion of proteins composed of low complexity protein sequence (LPS); number of proteins in an ortho-group (Ortho); presence of one of more Pfam domains (PFAM); Homology (E-value $1e^{-5}$) to a TrEMBL protein (TAXID); and transcriptional support for a gene model based on normalised read counts (transcripts per million, TPM). Panel **a**: First and second principal components of a PCA analysis using curated features; the clusters are coloured based on k-means clustering ($n = 7$). Panel **b**: Characteristics of each k-means cluster and the numbers of genes in individual clusters (left).

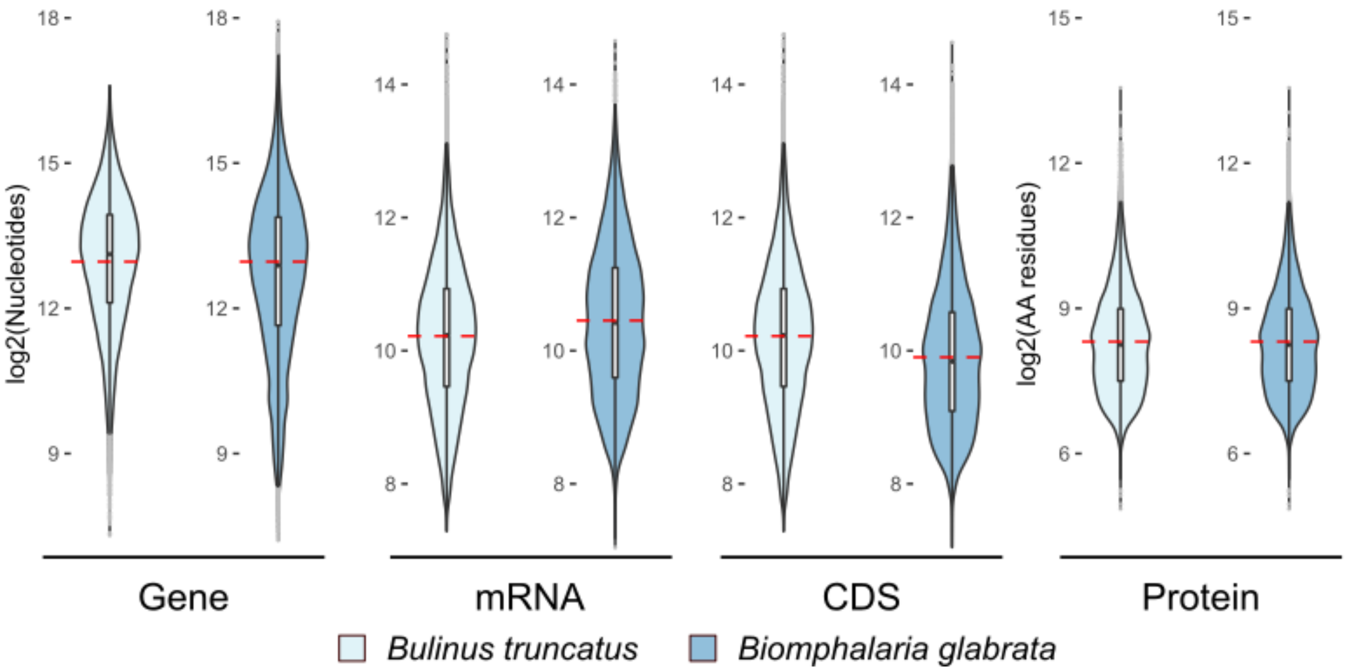

Supplementary Fig. 5 Gene model features. Violin and box plots comparing the lengths of genes, mRNAs, coding DNA sequences (CDSs) and proteins between *Bulinus truncatus* and *Biomphalaria glabrata* (BB02 strain). For each of the four features, 26,292 and 25,539 independent sequence lengths are summarised for each *B. truncatus* and *B. glabrata* plot, respectively, with each box plot indicating the interquartile range (IQR). The lower and upper boundaries of each box plot correspond to the first and third quartiles, respectively. Box plot whiskers are ±1.5 × IQR, with outliers indicated as grey points. The median (black line) and mean (red, dashed line) observations are shown.

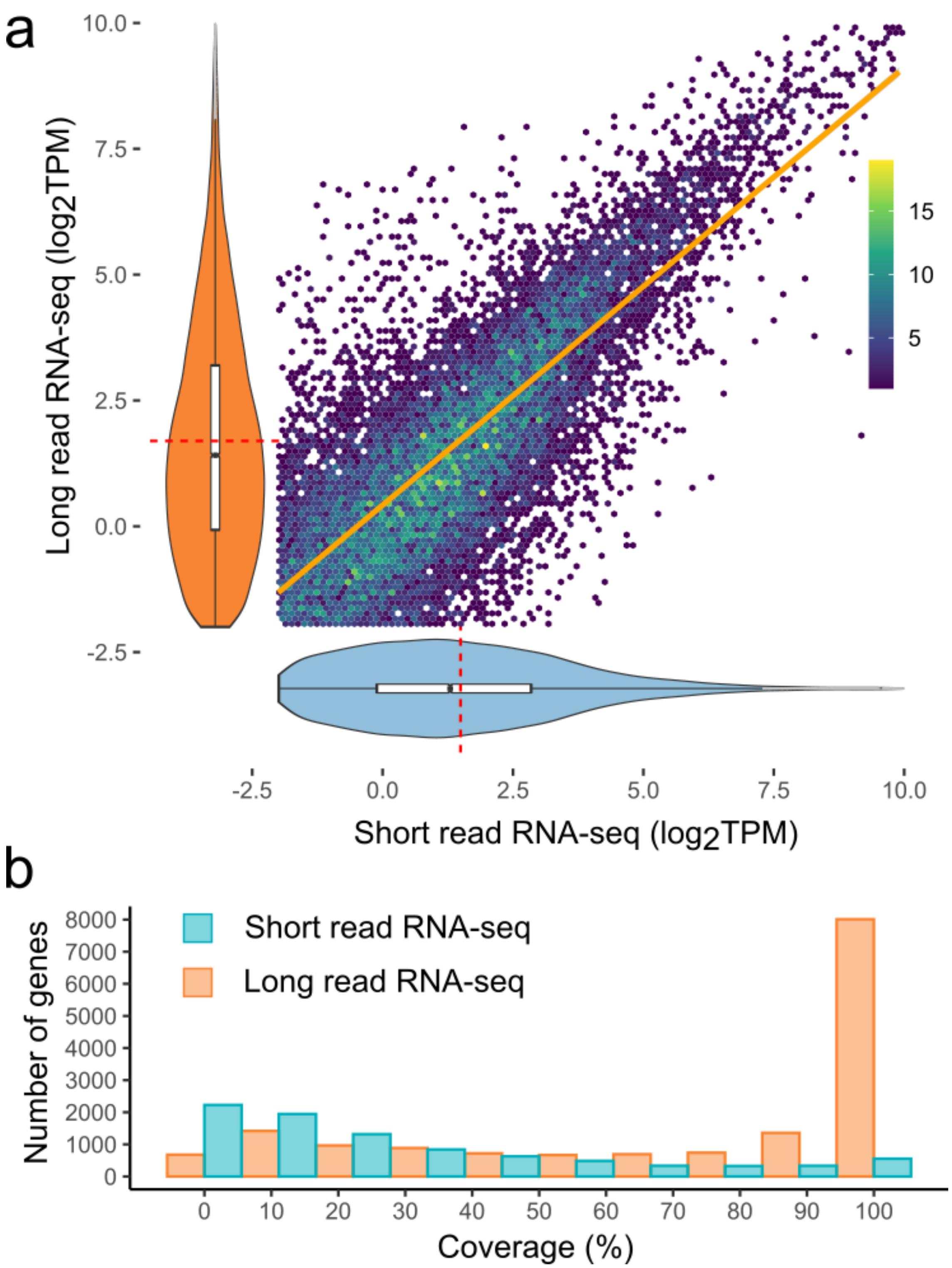

Supplementary Fig. 6 Evidence for gene models. Transcriptional support for 14,801 gene models for *Bulinus truncatus*, with levels of transcription (transcripts per million, TPM) values of > 0.2 TPM using data obtained from short-read (Illumina) and long-read (Oxford Nanopore) RNA-seq libraries Panel **a:** Correlation between short-read and long-read RNA-seq $\log_2$ TPM. The regression line inferred from linear regression analysis is shown (orange line; F-statistic: $2.859e^{+04}$ on 1 and 14799 degrees of freedom; adjusted $R^2 = 0.659$, p-value ≤ 0.001). Hex-bin plot is coloured based on gene density within the correlation plot. Violin and box plots show the distribution of long- and short-read lengths for 14,801 gene models. Each box plot indicates the interquartile range (IQR). The lower and upper boundaries of each box plot correspond to the first and third quartiles, respectively. Box plot whiskers are ±1.5 × IQR, with outliers indicated as grey points. The median (black line) and mean (red, dashed line) observations are shown. Panel **b**: Histogram showing the transcriptional coverage support for each predicted gene model.

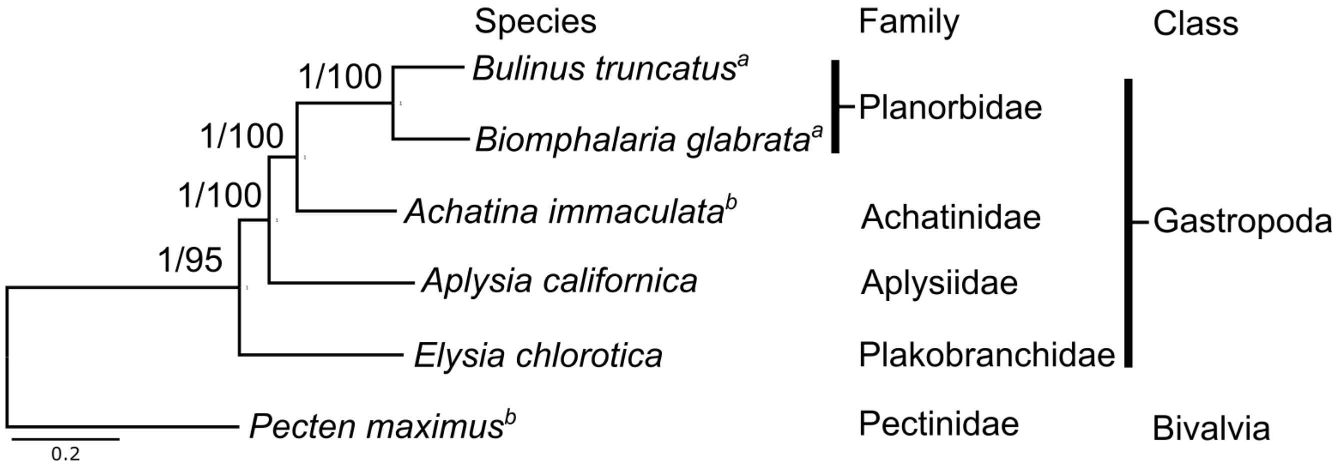

Supplementary Fig. 7 Phylogenetic position of *Bulinus truncatus* relative to other molluscs studied here. Trees constructed using Bayesian inference and maximum likelihood (ML) analyses of amino acid sequence data inferred from 2315 single-copy orthologs had the same topology. Nodal support values for BI and ML analyses are indicated at each branch (posterior probability and bootstrap support, respectively). Branch lengths represent the numbers of amino acid substitutions per site at aligned positions. [a]Snail species that act as intermediate hosts for schistosome parasites. [b] Molluscs for which chromosome-contiguous reference genomes are available. *Pecten maximus* (class Bivalvia) was selected as an outgroup.

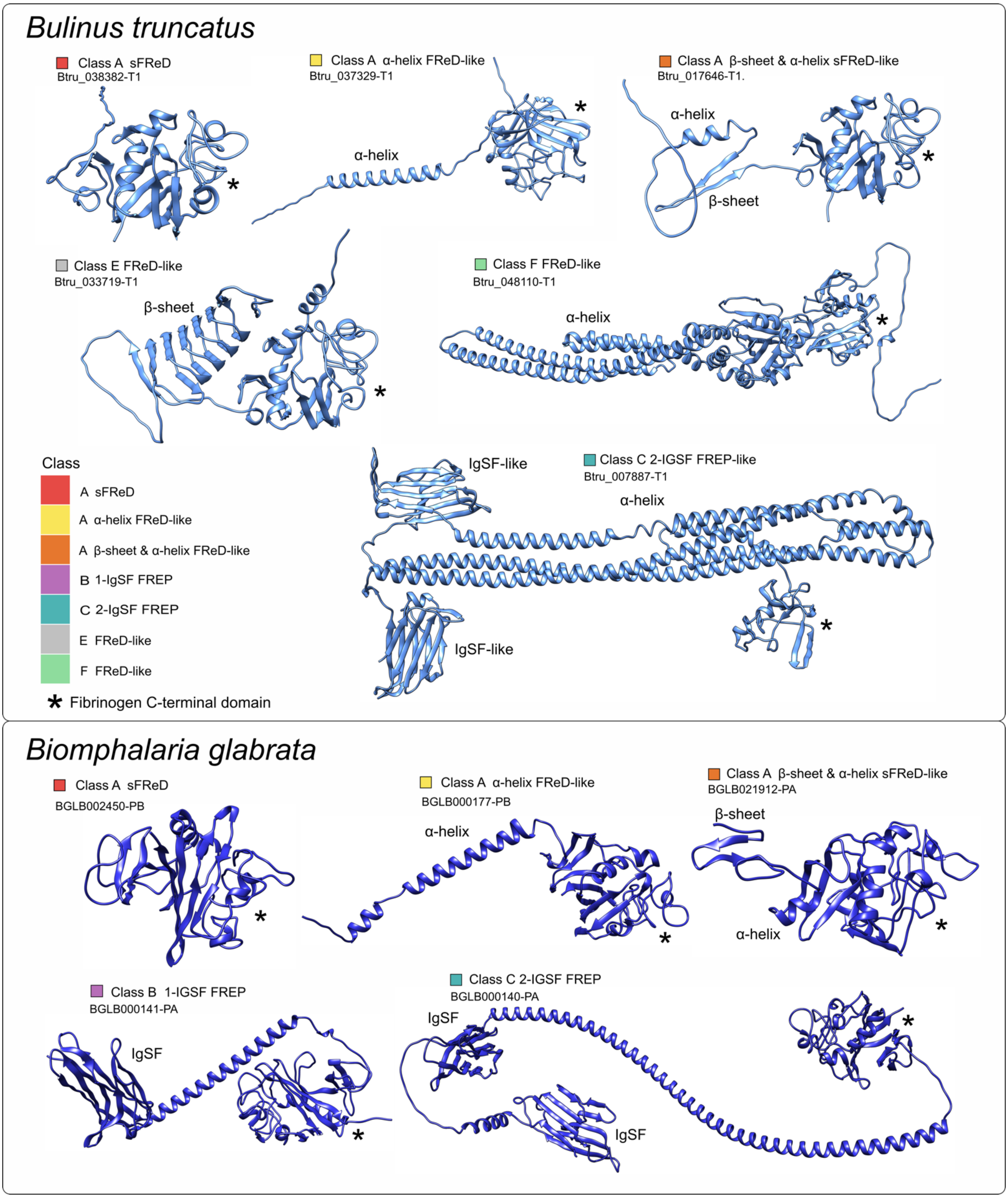

Supplementary Fig. 8 Structural classification of FReDs. Tertiary structure models for FReDs of *Bulinus truncatus* and *Biomphalaria glabrata* representing classes A–F. Fibrogen C-terminal domains (*), IgSF-like as well as α-helix and β-sheet structures are indicated.
