# Supplementary material for: Nuclear genome of Bulinus truncatus, an intermediate host of the carcinogenic human blood fluke Schistosoma haematobium
Source: Nat Commun. 2022 Feb 21;13:977. doi: 10.1038/s41467-022-28634-9 (PMC8861042; doi:10.1038/s41467-022-28634-9)
Supplement: Supplementary file 3 — Description of Additional Supplementary Files [file 41467_2022_28634_MOESM3_ESM.pdf]

Description of Additional Supplementary Files

File Name: Supplementary Data 1
Description: Evidence of transcription, and functional annotation of proteins inferred from genes in the genome (Btru.v1) of Bulinus truncatus – based on sequence similarity.

File Name: Supplementary Data 2
Description: KEGG protein family and pathway annotations of proteins inferred from genes in the genome (Btru.v1) of Bulinus truncatus – based on sequence similarity.

File Name: Supplementary Data 3
Description: Predicted tertiary structure models of classified fibrinogen-related lectins in the proteomes of Bulinus truncatus and Biomphalaria glabrata.
