# Supplementary material for: Nuclear genome of Bulinus truncatus, an intermediate host of the carcinogenic human blood fluke Schistosoma haematobium
Source: Nat Commun. 2022 Feb 21;13:977. doi: 10.1038/s41467-022-28634-9 (PMC8861042; doi:10.1038/s41467-022-28634-9)
Supplement: Supplementary file 7 — Reporting Summary [file 41467_2022_28634_MOESM7_ESM.pdf]

nature portfolio

Corresponding author(s): Neil Young
Last updated by author(s): Jan 14, 2022

Reporting Summary

Nature Portfolio wishes to improve the reproducibility of the work that we publish. This form provides structure for consistency and transparency in reporting. For further information on Nature Portfolio policies, see our Editorial Policies and the Editorial Policy Checklist.

Statistics

For all statistical analyses, confirm that the following items are present in the figure legend, table legend, main text, or Methods section.

n/a	Confirmed	
☐	☒	The exact sample size (*n*) for each experimental group/condition, given as a discrete number and unit of measurement
☐	☒	A statement on whether measurements were taken from distinct samples or whether the same sample was measured repeatedly
☐	☒	The statistical test(s) used AND whether they are one- or two-sided<br>*Only common tests should be described solely by name; describe more complex techniques in the Methods section.*
☒	☐	A description of all covariates tested
☒	☐	A description of any assumptions or corrections, such as tests of normality and adjustment for multiple comparisons
☐	☒	A full description of the statistical parameters including central tendency (e.g. means) or other basic estimates (e.g. regression coefficient) AND variation (e.g. standard deviation) or associated estimates of uncertainty (e.g. confidence intervals)
☐	☒	For null hypothesis testing, the test statistic (e.g. *F*, *t*, *r*) with confidence intervals, effect sizes, degrees of freedom and *P* value noted<br>*Give P values as exact values whenever suitable.*
☐	☒	For Bayesian analysis, information on the choice of priors and Markov chain Monte Carlo settings
☒	☐	For hierarchical and complex designs, identification of the appropriate level for tests and full reporting of outcomes
☒	☐	Estimates of effect sizes (e.g. Cohen's *d*, Pearson's *r*), indicating how they were calculated

Our web collection on statistics for biologists contains articles on many of the points above.

Software and code

Policy information about availability of computer code

Data collection: All platforms are cited in the Methods section and software and code is publicly available and cited correctly

Data analysis: Software used for data analysis, version controls and relevant citations or hyperlinks are listed below

General software
R v.3.4.3 (http://www.R-project.org/) was used for creating summary statistics and running several cited packages.

Software used in sequencing
Guppy v.4.2.2 (Oxford Nanopore Technologies) (https://community.nanoporetech.com/downloads)

Software used for genome assembly
FLYE v2.8-b1674 (Kolmogorov et al., 2019) used for genome assembly
medaka package v.1.0.3 (https://github.com/nanoporetech/medaka) used to correct nanopore sequence errors using medaka_consensus
pilon v.1.23 (Walker et al., 2014) was used to polish contigs using short-insert (500 bp) genomic DNA library
3D-DNA v.180922 (Dudchenko et al., 2017) was used to scaffold contigs using Hi-C
purge_haplotigs v.1.1.1 (Roach et al., 2018) was used to remove haplotig redundancy
TGS-GapCloser v.1.1.1 (https://github.com/BGI-Qingdao/TGS-GapCloser) was used to close gaps in scaffolds
BUSCO v 4.0.2 (Simao et al., 2015) was used to assess the completeness of the genome (in genome-mode)

Software used to assess genome size, heterozygosity and ploidy
kmc v.3.1.1 (Kokot et al., 2017) was used to create 21-mer frequency histograms using the short-insert (500 bp) genomic DNA library
GenomeScope v.2.0 and smudgeplot v.0.2.4 packages (Ranallo-Benavidez et al., 2020) was used to estimate genome size, heterozygosity and

ploidy
bwa v.2 (Li and Durbin, 2009) was used to map short-insert genomic DNA library to the reference genome
PloiPy https://github.com/floutt/PloidPy was used to estimate minor allelic frequencies and ploidy using mapped DNA library BAM file.

Software used to predict repeat-elements
RepeatModeler v. 1.0.8 (http://www.repeatmasker.org) was used to predict and annotate repeat elements in the genome
EDTA v v.1.9.4 (Ou et al., 2019) was used to predict and annotate repeat elements in the genome
CD-HIT v.4.8.1 (Fu et al., 2012) was used to combine repeat libraries and remove redundancy
RepeatMasker v.4.1.1 (Tarailo-Graovac and Chen, 2009) was used to mask the genome using the final repeat element library

Software used to predict gene models
funannotate v.1.7.4 (https://github.com/nextgenusfs/funannotate) was used to predict gene models
evidence modeler (EVM) v.1.1.1 (Haas et al., 2008) was used to weigh and select gene models inferred from multiple sources.
PASA v.2.4.1 (Haas et al., 2008) was used to predict gene models
augustus v.3.3.3 (Hoff and Stanke, 2019) was used to predict gene models
StringTie v2.1.2 (Pertea et al., 2015) was used to predict gene models
geneMark ES v.3.32 (Lomsadze et al., 2005) was used to predict gene models
BUSCO v 4.0.2 (Simao et al., 2015) was used to assess the completeness of the gene set (in protein-mode)

Software used to predict protein function.
InterPro v5.35 (Zdobnov and Apweiler, 2001) was used to infer conserved domains within predicted proteins
EggNOG mapper v.5.0 (Huerta-Cepas et al., 2019) was used to infer function of predicted proteins
DIAMOND BLASTp v. 0.9.21 (Buchfink et al., 2015) was used to identify homology to predicted proteins within accessed sequence databases.
phobius v.1.04 (Kall et al., 2007) was used to predict signal peptides and transmembrane domains
MultiLoc2 v.2.2.25 (Blum et al., 2009) was used to predict the sub-cellular localisation of protein sequences
HISAT2 v.2.1.0 (Kim et al., 2019) was used to map short and long RNA-seq data to the genome
StringTie v2.1.2 (Pertea et al., 2015) was used to infer levels of transcription per gene (in transcripts per million, TPM) from mapped RNA-seq reads
GeneValidator v.2.1.10 (Dragan et al., 2016) was used to score genes based on comparisons to proteins in Swiss-Prot within UniProtKB
fLPS v.1 (Harrison, 2017) was used to estimate the proportion of proteins predicted to be a “low probability subsequence” (LPS)
OrthoFinder v.2.3.11 (Emms and Kelly, 2019) was used to estimate individual groups of orthologous protein between snail species included in this study
Exonerate v.2.0 (Slater and Birney, 2005) was used to create a genome feature format file of the location of orthologous proteins within snail genomes.
hmmsearch (HMMER v.3.2.1; http://hmmer.janelia.org/) was used to
AlphaFold v1 (Jumper et al., 2021) was used to predict tertiary structures from mature peptide sequences.
circos v.0.69-8 (Krzywinski et al., 2009) was used to render images with locations of paired single copy orthologues in the same genomic region of each genome

Software used for phylogenetic analyses
MUSCLE v3.8.31 (Edgar, 2004) was used to align clusters of single-copy orthologues proteins
MAFFT v.7.271 (Katoh et al., 2005) was used to align clusters of single-copy orthologues proteins
AQUA (Muller et al., 2010) was used to combine results from MUSCLE and MAFFT alignment software
RASCAL v1.34 (Thompson et al., 2003) was used to refine sequence alignments
NorMD (Thompson et al., 2001) v1 was use to infer sequence alignment scores
PartitionFinder v2.1.1 (Lanfear et al., 2017) was used to infer optimal amino acid substitution matrices and merge sequences into simimilar partitions
MrBayes v3.2.6 (Ronquist and Huelsenbeck, 2003) was used for Bayesian inference (BI) analyses
RAxML v8.2.6 (Stamatakis et al., 2005) was used for maximum likelihood (ML) tree-building analyses
FigTree v.1.31 (http://tree.bio.ed.ac.uk/software/figtree). was used to prepare representative phylogenetic trees
ggtree (v.1.10.5) (Yu et al., 2017) was used to render and annotate phylogenetic trees

Citations for publicly available software

Blum T., Briesemeister S., Kohlbacher O., 2009. MultiLoc2: integrating phylogeny and Gene Ontology terms improves subcellular protein localization prediction. BMC Bioinformatics 10, 274.
Buchfink B., Xie C., Huson D.H., 2015. Fast and sensitive protein alignment using DIAMOND. Nat. Methods 12, 59-60.
Dragan M.A., Moghul I., Priyam A., Bustos C., Wurm Y., 2016. GeneValidator: identify problems with protein-coding gene predictions. Bioinformatics 32, 1559-1561.
Dudchenko O., Batra S.S., Omer A.D., Nyquist S.K., Hoeger M., Durand N.C., et al., 2017. De novo assembly of the Aedes aegypti genome using Hi-C yields chromosome-length scaffolds. Science 356, 92-95.
Edgar R.C., 2004. MUSCLE: a multiple sequence alignment method with reduced time and space complexity. BMC Bioinformatics 5, 113.
Emms D.M., Kelly S., 2019. OrthoFinder: phylogenetic orthology inference for comparative genomics. Genome Biol. 20, 238.
Fu L., Niu B., Zhu Z., Wu S., Li W., 2012. CD-HIT: accelerated for clustering the next-generation sequencing data. Bioinformatics 28, 3150-3152.
Haas B.J., Salzberg S.L., Zhu W., Pertea M., Allen J.E., Orvis J., et al., 2008. Automated eukaryotic gene structure annotation using EvidenceModeler and the program to assemble spliced alignments. Genome Biol. 9, R7.
Harrison P.M., 2017. fLPS: Fast discovery of compositional biases for the protein universe. BMC Bioinformatics 18, 476.
Hoff K.J., Stanke M., 2019. Predicting genes in single genomes with AUGUSTUS. Curr. Protoc. Bioinformatics 65, e57.
Huerta-Cepas J., Szklarczyk D., Heller D., Hernandez-Plaza A., Forslund S.K., Cook H., et al., 2019. eggNOG 5.0: a hierarchical, functionally and phylogenetically annotated orthology resource based on 5090 organisms and 2502 viruses. Nucleic Acids Res. 47, D309-D314.

Jumper J., Evans R., Pritzel A., Green T., Figurnov M., Ronneberger O., et al., 2021. Highly accurate protein structure prediction with AlphaFold. Nature 596, 583-589.
Kall L., Krogh A., Sonnhammer E.L., 2007. Advantages of combined transmembrane topology and signal peptide prediction--the Phobius web server. Nucleic Acids Res. 35, W429-432.
Katoh K., Kuma K., Toh H., Miyata T., 2005. MAFFT version 5: improvement in accuracy of multiple sequence alignment. Nucleic Acids Res. 33, 511-518.
Kim D., Paggi J.M., Park C., Bennett C., Salzberg S.L., 2019. Graph-based genome alignment and genotyping with HISAT2 and HISAT-genotype. Nat. Biotechnol. 37, 907-915.
Kokot M., Dlugosz M., Deorowicz S., 2017. KMC 3: counting and manipulating k-mer statistics. Bioinformatics 33, 2759-2761.
Kolmogorov M., Yuan J., Lin Y., Pevzner P.A., 2019. Assembly of long, error-prone reads using repeat graphs. Nat. Biotechnol. 37, 540-546.
Krzywinski M., Schein J., Birol I., Connors J., Gascoyne R., Horsman D., et al., 2009. Circos: an information aesthetic for comparative genomics. Genome Res. 19, 1639-1645.
Lanfear R., Frandsen P.B., Wright A.M., Senfeld T., Calcott B., 2017. PartitionFinder 2: New Methods for Selecting Partitioned Models of Evolution for Molecular and Morphological Phylogenetic Analyses. Mol. Biol. Evol. 34, 772-773.
Li H., Durbin R., 2009. Fast and accurate short read alignment with Burrows-Wheeler transform. Bioinformatics 25, 1754-1760.
Lomsadze A., Ter-Hovhannisyan V., Chernoff Y.O., Borodovsky M., 2005. Gene identification in novel eukaryotic genomes by self-training algorithm. Nucleic Acids Res. 33, 6494-6506.
Muller J., Creevey C.J., Thompson J.D., Arendt D., Bork P., 2010. AQUA: automated quality improvement for multiple sequence alignments. Bioinformatics 26, 263-265.
Ou S., Su W., Liao Y., Chougule K., Agda J.R.A., Hellinga A.J., et al., 2019. Benchmarking transposable element annotation methods for creation of a streamlined, comprehensive pipeline. Genome Biol. 20, 275.
Pertea M., Pertea G.M., Antonescu C.M., Chang T.C., Mendell J.T., Salzberg S.L., 2015. StringTie enables improved reconstruction of a transcriptome from RNA-seq reads. Nat. Biotechnol. 33, 290-295.
Ranallo-Benavidez T.R., Jaron K.S., Schatz M.C., 2020. GenomeScope 2.0 and Smudgeplot for reference-free profiling of polyploid genomes. Nat. Commun. 11, 1432.
Roach M.J., Schmidt S.A., Borneman A.R., 2018. Purge Haplotigs: allelic contig reassignment for third-gen diploid genome assemblies. BMC Bioinformatics 19, 460.
Ronquist F., Huelsenbeck J.P., 2003. MrBayes 3: Bayesian phylogenetic inference under mixed models. Bioinformatics 19, 1572-1574.
Simao F.A., Waterhouse R.M., Ioannidis P., Kriventseva E.V., Zdobnov E.M., 2015. BUSCO: assessing genome assembly and annotation completeness with single-copy orthologs. Bioinformatics 31, 3210-3212.
Slater G.S., Birney E., 2005. Automated generation of heuristics for biological sequence comparison. BMC Bioinformatics 6, 31.
Stamatakis A., Ludwig T., Meier H., 2005. RAxML-III: a fast program for maximum likelihood-based inference of large phylogenetic trees. Bioinformatics 21, 456-463.
Tarailo-Graovac M., Chen N., 2009. Using RepeatMasker to identify repetitive elements in genomic sequences. Curr. Protoc. Bioinformatics Chapter 4, Unit 4 10.
Thompson J.D., Plewniak F., Ripp R., Thierry J.C., Poch O., 2001. Towards a reliable objective function for multiple sequence alignments. J. Mol. Biol. 314, 937-951.
Thompson J.D., Thierry J.C., Poch O., 2003. RASCAL: rapid scanning and correction of multiple sequence alignments. Bioinformatics 19, 1155-1161.
Walker B.J., Abeel T., Shea T., Priest M., Abouelliel A., Sakthikumar S., et al., 2014. PILON: an integrated tool for comprehensive microbial variant detection and genome assembly improvement. PLoS ONE 9, e112963.
Yu G.C., Smith D.K., Zhu H.C., Guan Y., Lam T.T.Y., 2017. GGTREE: an R package for visualization and annotation of phylogenetic trees with their covariates and other associated data. Methods Ecol. Evol. 8, 28-36.
Zdobnov E.M., Apweiler R., 2001. InterProScan--an integration platform for the signature-recognition methods in InterPro. Bioinformatics 17, 847-848.

For manuscripts utilizing custom algorithms or software that are central to the research but not yet described in published literature, software must be made available to editors and reviewers. We strongly encourage code deposition in a community repository (e.g. GitHub). See the Nature Portfolio guidelines for submitting code & software for further information.

Data

Policy information about availability of data

All manuscripts must include a data availability statement. This statement should provide the following information, where applicable:

- Accession codes, unique identifiers, or web links for publicly available datasets
- A description of any restrictions on data availability
- For clinical datasets or third party data, please ensure that the statement adheres to our policy

The nucleotide sequence data linked to the nuclear genome reported in this article is publicly available in the GenBank database and the Sequence Read Archive (SRA) under the accession numbers SAMN17050146, SAMN16898649 and SAMN16898648 with the NCBI BioProject accession number PRJNA680620. Protein sequences used for sequence homology searches are available from the Swiss-Prot (UniProtKB ;accessed 20 December 2020) [25], TrEMBL (UniProtKB; accessed 20 December 2020) [25] , Encyclopedia of Genes and Genomes (KEGG) (accessed 20 December 2020) [79] and MEROPS release 12 [80] databases. All other data used are referred to in this article and its supplementary files.

Field-specific reporting

Life sciences study design

All studies must disclose on these points even when the disclosure is negative.

Sample size	Samples were obtained from an inbred lines for snail maintained for research purposes. No sample size was chosen as the genome was sequenced to create a reference genome for an inbred laboratory line. This one genome is not meant to represent all genotypes of Bulinus truncatus in Africa
Data exclusions	No data was excluded
Replication	Replication was not required to complete the analyses herein. No treatment and control-type analyse were performed herein that would required a measure of biological variation among individual snails. Measures of transcription are only reported with no differences in levels of transcription mentioned.
Randomization	Not applicable. This was an inbred laboratory strain with little to no genetic variation present that would impact our results.
Blinding	Not applicable. No results presented are susceptible to user interpretation.

Reporting for specific materials, systems and methods

We require information from authors about some types of materials, experimental systems and methods used in many studies. Here, indicate whether each material, system or method listed is relevant to your study. If you are not sure if a list item applies to your research, read the appropriate section before selecting a response.

Materials & experimental systems

n/a	Involved in the study	
☒	☐	Antibodies
☒	☐	Eukaryotic cell lines
☒	☐	Palaeontology and archaeology
☐	☒	Animals and other organisms
☒	☐	Human research participants
☒	☐	Clinical data
☒	☐	Dual use research of concern

Methods

n/a	Involved in the study	
☒	☐	ChIP-seq
☒	☐	Flow cytometry
☒	☐	MRI-based neuroimaging

Animals and other organisms

Policy information about studies involving animals; ARRIVE guidelines recommended for reporting animal research

Laboratory animals	Adult specimens of Bulinus truncatus (5- to 7-week-old; hermaphrodites) originated from a laboratory line (designated 'BRI'), which is routinely maintained in the Biomedical Research Institute (BRI), Rockville, MD, USA
Wild animals	No wild animals were used.
Field-collected samples	No animals were collected from the field
Ethics oversight	No ethics was required to maintain this snail in the laboratory.

Note that full information on the approval of the study protocol must also be provided in the manuscript.
